# Molecular Fractionation Induced by Viscosity-Driven Segregative Phase Separation Behavior of Gum Arabic/Hydroxypropyl Methylcellulose

**DOI:** 10.3390/foods14152642

**Published:** 2025-07-28

**Authors:** Lingyu Han, Cunzhi Zhang, Nuo Dong, Jixin Yang, Qiuyue Zheng, Xiaobo Zhang, Ronggang Liu, Jijuan Cao, Bing Hu

**Affiliations:** 1 Key Lab of Biotechnology and Bioresources Utilization of Ministry of Education, College of Life Science, Dalian Minzu University, Dalian 116600, China; hlbehu@163.com (C.Z.); dongnuo931529@163.com (N.D.); zhengqy@dlnu.edu.cn (Q.Z.); zhangxb2023@dlnu.edu.cn (X.Z.); lrg19950427@163.com (R.L.); 20191414@dlnu.edu.cn (J.C.); 2 Faculty of Social and Life Sciences, Wrexham University, Plas Coch, Mold Road, Wrexham LL11 2AW, UK; jixin.yang@wrexham.ac.uk

**Keywords:** segregative phase separation, viscosity, fractionation, gum arabic, hydroxypropyl methyl cellulose

## Abstract

Segregative phase separation technology demonstrates substantial potential for precise molecular fractionation in food and biomaterial applications. The investigation elucidates the causal relationship between viscosity variations and phase separation dynamics, which govern molecular fractionation in GA/HPMC composite systems. By conducting a comparative analysis of two GA subtypes (CGA and SGA) and three HPMC grades with controlled viscosity gradients, we utilized gel permeation chromatography-multi-angle laser light scattering (GPC-MALLS) coupled with rheological characterization to elucidate the critical relationship between continuous phase viscosity and fractionation efficiency. Notably, increasing HPMC viscosity significantly intensified phase separation, resulting in selective enrichment of arabinogalactan-protein complexes: from 6.3% to 8.5% in CGA/HPMC systems and from 27.3% to 36.5% in SGA/HPMC systems. Further mechanistic investigation revealed that elevated HPMC viscosity enhances thermodynamic incompatibility while slowing interfacial mass transfer, synergistically driving component redistribution. These findings establish a quantitative viscosity–fractionation relationship, offering theoretical insights for optimizing GA/HPMC systems in emulsion stabilization, microencapsulation, and functional biopolymer purification via viscosity-mediated phase engineering.

## 1. Introduction

Hydrocolloids, a group of polymers with excellent hydrophilicity, are commonly used as food additives to manipulate the texture, microstructure, flavor, and shelf life of food products. Most hydrocolloids are natural biopolymers and therefore have a complex composition, which results in typical polydispersity characteristics [1]. Traditional hydrocolloids such as gum arabic (GA), xanthan gum (XG), and carrageenan are widely used in the food, pharmaceutical, and cosmetic industries owing to their excellent emulsification properties, stability, water holding capacity, and thickening ability [2,3,4]. With technological advancements, the demand for hydrocolloids with improved performance characteristics has expanded. However, traditional hydrocolloids have failed to meet these requirements, and several new types of colloids have been developed. Additionally, novel methods for the applications of various colloids have also been established [5].

When two or more natural polymers are blended together, serious incompatibilities can occur under certain conditions. This results in phase separation—a phenomenon crucial for industrial applications such as the structural design of foods, preparation of multiple emulsions, micro-encapsulation, and protein purification and separation [6,7,8]. The separation of biopolymer pairs within a specific liquid phase can be classified into two categories—associative phase separation and segregative phase separation—but most studies so far have only focused on the former technique. Notably, associative phase separation, also known as composite coalescence, occurs via the merging of two oppositely charged macromolecules. This process is primarily driven by Coulombic electrostatic forces and the entropy elevations resulting from the release of counter-ions [1,9]. Segregative phase separation between biopolymers results from either effective electrostatic repulsion or asymmetric biopolymer-solvent interactions. The self-segregation dynamics in mutually incompatible polymer systems generally proceed through two distinct stages: initial phase separation leading to the formation of component-enriched domains, followed by molecular sorting wherein polymers selectively migrate based on their structural parameters. While the mechanisms driving associative and segregative phase separations differ, these processes can occur simultaneously in some case [10].

Gum arabic (GA) is the natural exudate produced by the bark of acacia trees, which serve as the source for 80% of the global production of commercial GA. GA has long served as a multifunctional additive in industrial manufacturing processes, with its primary roles centered on viscosity modification and emulsion stabilization. The three main components of GA are arabinogalactan-protein complex (AGP), arabinogalactan (AG), and glycoprotein (GP) [11]. Of these components, AGP has a protein content of approximately 10%, and its molecular mass can reach a few million Daltons. Moreover, it is generally believed that AGP is a key contributor to the emulsification properties of GA. In this study, two types of GA were studied: the commercially used common gum arabic (CGA) and super gum arabic (SGA, also known as EM10). SGA is an enhanced gum arabic obtained by maturation treatment, during which the naturally occurring association of low-molecular-weight arabinogalactan (AG) and glycoprotein (GP) units progresses further to form larger arabinogalactan-protein (AGP) aggregates. Consequently, SGA exhibits a higher AGP content and increased average molecular weight [12].

Hydroxypropyl methylcellulose (HPMC) is a mixed cellulose ether in which the hydroxypropyl and methyl groups are chemically modified and linked to the β-1,4-D dextran backbone to generate a white to off-white powder. HPMC is highly soluble in water, which makes it an excellent choice for aqueous formulations. It exhibits excellent hydrophilicity, solubility, and film-forming properties, enabling it to function effectively as a thickener, emulsifier, and stabilizer. HPMC is widely applied in the food industry, particularly as a structurally stable polysaccharide for the preparation of edible films [13,14]. Recent research has revealed that HPMC is a unique polysaccharide and possesses a remarkable range of functions across various biological processes. For instance, Zhou et al. investigated the effect of HPMC on the hardening of high-protein nutritional bars and found that HPMC could serve as an inhibitor, suppressing the Meladic reaction and preventing protein aggregation during food storage [15].

Although the thermodynamic parameters and molecular characteristics that govern phase separation have been extensively elucidated, the hydrodynamic control exerted by viscosity remains significantly underexplored within the current phase separation frameworks [12,16]. At the molecular level, increased solution viscosity enhances hydrodynamic resistance, which reduces the diffusion coefficients of polymer chains by Stokes–Einstein dynamic suppression, and alters the critical polymer concentration required for phase separation through excluded volume effects [17]. Specifically, in polydisperse GA/HPMC systems, higher HPMC viscosity creates a steric hindrance network that preferentially restricts the mobility of larger AGP complexes, forcing their selective partitioning into the polymer-rich phase while allowing smaller AG/GP components to migrate toward the dilute phase—a phenomenon termed viscosity-driven molecular sieving [18].

The objective of this paper is to investigate the effect of viscosity on the segregative phase separation of gum arabic (GA) and hydroxypropyl methylcellulose (HPMC) systems, focusing on the molecular fractionation and the resulting distribution of arabinogalactan-protein complexes (AGP). By manipulating the viscosity of HPMC solutions, we aim to elucidate how viscosity modulates phase separation dynamics, thus contributing to the development of more efficient fractionation processes in food and biomaterial systems [19].

## 2. Materials and Methods

### 2.1. Materials

The experimental materials included two GA variants: SGA (analytical reagent grade, ≥99% purity, supplied by San Ei Gen F.F.I, Osaka, Japan; powder form, LOT 101008) and CGA (ACS-certified, provided by Aladdin, Shanghai, China; powder form, LOT E2008126). Both were characterized as hygroscopic white powders. Advanced GPC analysis revealed distinct macromolecular profiles: SGA exhibited a highly polydisperse architecture with a weight-average molecular weight (Mw) of 2.83 MDa, while CGA displayed quasi-monodispersity with an Mw of 600 kDa. Complementary HPMC (ultra-pure grade, 99.5%, Aladdin A2206761) was employed as the polymeric modulator. Notably, 100 mPa·s HPMC had a Mw of 7.3 × 10^4^ g/mol and a Mw/Mn value of 1.5; 15,000 mPa·s HPMC, Mw of 3.3 × 10^5^ g/mol and a Mw/Mn value of 1.3; and 100,000 mPa·s HPMC, Mw of 4.8 × 10^5^ g/mol and a Mw/Mn value of 1.2. The molecular weight values were determined using a gel permeation chromatography-multi-angle laser light scattering (GPC-MALLS) system, as described previously [20].

### 2.2. Preparation of Stock Solutions

Working solutions of CGA and SGA (30 wt% each) were prepared by dispersing their respective lyophilized powders in deionized water supplemented with 0.005 wt% sodium azide as a preservative. The resulting suspensions were subjected to continuous rotational mixing (SRT-303, Haimen Kylin-Bell Lab Instruments Co., Ltd., Haimen, China) at 150 r/min for 16 h at 25 °C to ensure complete hydration equilibrium [21]. For HPMC preparation, 2 wt% anhydrous powder was dispersed in the same azide-containing aqueous medium, followed by thermal homogenization at 80 °C for 30 min and subsequent extended drum mixing for 24 h at ambient temperature to achieve full polymer solvation [22]. Unless otherwise specified, all concentrations in this study are expressed on a weight/weight basis.

### 2.3. Preparation of GA/HPMC Mixed Aqueous Solutions

Prepare 10 g of a 6% CGA/1% HPMC aqueous solution containing 0.06 g CGA and 0.01 g HPMC, and 10 g of an 8% SGA/1% HPMC aqueous solution containing 0.08 g SGA and 0.01 g HPMC. The selection of the ratio between CGA or SGA and HPMC is based on the phase diagram, ensuring that all mixed solutions exhibit segregative phase separation at the same concentration. All mixed solutions were homogenized for 5 min using a vortex mixer (MX-S, DLAB Scientific Co., Ltd., Beijing, China). These solutions were prepared using HPMC of different viscosities (100 mPa·s, 15,000 mPa·s, and 100,000 mPa·s) and stock solutions of two types of GA. The mixtures were centrifuged (CH210R, Hunan Xiangyi Laboratory Instrument Development Co., Ltd., Changsha, China) at 25 °C and 4000 rpm for 3 h.

### 2.4. Zeta Potential Measurements

The zeta potential values of GA were determined using a Zetasizer Nano-ZSP system (Malvern Instruments Ltd., Worcestershire, UK) equipped with laser doppler velocimetry and phase analysis light scattering. GA stock solutions were all diluted to a concentration of 0.05% *w*/*v* and then placed into disposable folded capillary cells (DTS1070) to measure the zeta potential at 25 °C based on the movement and velocity of the molecules under the applied electric field [23]. The average values of three replicate measurements were obtained.

### 2.5. GPC-MALLS

A comprehensive analytical methodology utilizing GPC-MALLS (Wyatt Technology, Santa Barbara, CA, USA) technology [23] was employed to assess the molecular characteristics of CGA, SGA, and three HPMC formulations, as well as the compositions of phase-separated systems at 25 °C. The mobile phase was prepared as a 0.2 M NaCl aqueous solution containing 0.005% sodium azide, filtered through a 0.2 μm membrane. Chromatographic separations were performed at a constant flow rate of 0.4 mL/min using a KNAUER K-501 HPLC pump (Kinesis, Berlin, Germany). Sample aliquots were introduced into the system after dilution and filtration through a 0.45 mm nylon membrane [21]. All experimental data were acquired and analyzed using Astra software (v4.90.08, Wyatt Technology, USA).

### 2.6. Determination of Separation Phase Diagram

The methodology utilized to construct separation phase diagrams for GA/HPMC systems through visual inspection techniques. Two GA derivatives (CGA and SGA) were individually mixed with HPMC solutions of three viscosity levels (100, 15,000, and 100,000 mPa·s), resulting in either homogeneous or phase-separated systems. Phase separation was operationally identified by the onset of turbidity either immediately or shortly after mixing, followed by distinct layer formation upon centrifugation [24,25]. Primary solutions of GA and HPMC were blended at diverse ratios to establish separation phase diagrams. Centrifugation conditions were set at 25 °C, with a rotational speed of 4000 rpm for 60 min (CH210R, Hunan Xiangyi Laboratory Instrument Development Co., Ltd., Changsha, China). Prolonged centrifugation beyond this duration did not significantly affect phase separation results. In complementary experimental series, HPMC concentrations were fixed within the range of 0.1–2.0%, while CGA/SGA concentrations were systematically varied between 2.0% and 30.0%. Subsequently, cloud points in the CGA(SGA)/HPMC systems were identified through macroscopic analysis of phase boundaries following centrifugation.

### 2.7. Rheological Analyses

Rheological analysis was performed using an AR 500 rheometer (TA Instruments, Staffordshire, UK), fitted with parallel plates (40 mm diameter) and configured with a 2000 μm inter-plate gap. The samples were placed on the plate of the rheometer at a test temperature of 25 °C, with an approximate sample volume of 2–3 g, and the shear viscosity was measured at shear rates between 0.01 and 100 s^−1^, following established protocols [23,24].

### 2.8. Statistical Analysis

All experimental procedures were performed in triplicate using independent replicates. Statistical analysis was conducted via one-way ANOVA, complemented by post hoc testing, with results presented as mean values ± standard deviation. Data processing was executed using IBM SPSS Statistics (Version 28.0, Armonk, NY). For all comparative evaluations, a significance level of *p* < 0.05 was adopted.

## 3. Results

### 3.1. Characterisation of GA and HPMC

**Figure 1:**
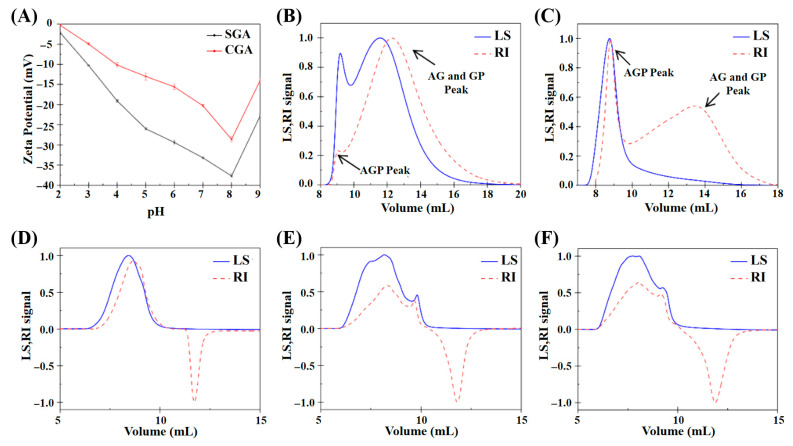
(**A**) Zeta potential values of SGA and CGA under different pH conditions. (**B**) Elution profile of CGA based on GPC-MALLS analysis. (**C**) Elution profile of SGA based on GPC-MALLS analysis. (**D**) Elution profile of 100 mPa·s HPMC based on GPC-MALLS analysis. (**E**) Elution profile of 15,000 mPa·s HPMC based on GPC-MALLS analysis. (**F**) Elution profile of 100,000 mPa·s HPMC based on GPC-MALLS analysis.

The zeta potential values of CGA and SGA were measured to understand their surface charge characteristics in aqueous solutions. Figure 1A shows both CGA and SGA exhibited negative zeta potentials, indicating that they carried negative surface charges within the measured pH range (2–9). However, it is important to note that zeta potential values represent the electric potential difference across the particle interface rather than the actual charge of the molecules. This electric potential differential is influenced by factors such as the ionic strength and pH of the medium, which can affect the distribution of ions around the particles. As the pH increased, both CGA and SGA exhibited a higher negative charge, likely due to the dissociation of carboxyl groups on the biopolymers’ surface, which is in agreement with findings from previous studies [12,26]. Figure 1B,C shows the plots of the refractive index (RI) and light scattering (LS) signals of CGA and SGA measured using GPC-MALLS analysis. The peaks of AGP and AG+GP can be detected on the RI signal curve of GPC-MALLS [21,27]. Figure 1D–F shows the plots of the RI and LS signals obtained from HPMC solutions of different viscosities. A clear molecular weight distribution was observed at an outflow volume of 5 to 10 mL. While the RI signal showed an inverted peak after 10 mL, the LS signal did not show any peak. This indicated that the solvent peak between 10 and 15 mL did not affect the overall experimental results.

The molecular parameters of the five samples (CGA, SGA, and 100, 15,000, and 100,000 mPa·s HPMC) were obtained based on RI and LS signal processing (Table 1).

Using GPC-MALLS, Tipvarakarnkoon et al. reported that the Mw of whole gum and the AGP fraction of GA were 3.43 × 10^6^ Da and 11.67 × 10^6^ Da, respectively [26]. These values are comparable to the results obtained in the present study. Our findings thus show that a greater proportion of the AGP fraction can be obtained via the maturation of GA. GA can be modified in the dry state in a range of physical forms, in a dry stainless-steel container or on a suitable surface, open to air or in a non-oxidizing environment (an atmosphere of nitrogen). The treatment involves maturation under strictly controlled conditions of temperature and humidity of the dry gum [28,29]. SGA is an enhanced form of gum arabic produced through a maturation process, during which the natural association between low-molecular-weight arabinogalactan (AG) and glycoprotein (GP) components further develops, leading to the formation of larger arabinogalactan-protein (AGP) complexes. As a result, SGA demonstrates an elevated AGP content and a higher average molecular weight. The results show that the molecular weight of AGP in SGA was found to be about 6.6 × 10^6^ Da, which is higher than the value of 1.52 × 10^6^ Da typically reported for CGA [20,30]. This indicated that the maturation process leads to an increase in the fraction of AGP in GA through the aggregation of AG and GP and, consequently, an increase in the average molecular weight, which is also reflected by the increase in the Rg value. Additionally, the data for HPMC revealed that both the molecular weight and Rg of HPMC increase significantly with its viscosity [31].

### 3.2. Viscosity-Dominated Phase Boundary Evolution

**Figure 2:**
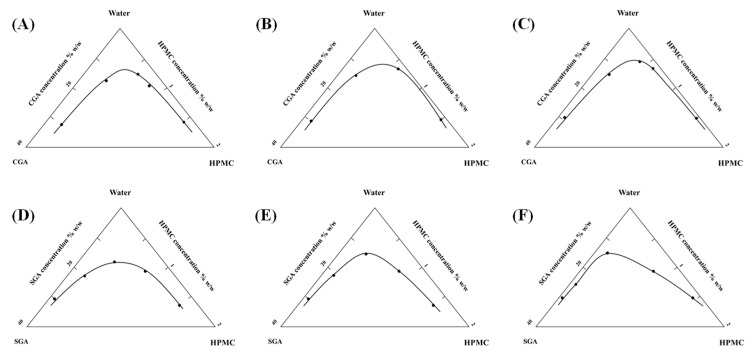
(**A**–**C**) Separation phase diagrams of CGA and 100, 15,000, and 100,000 mPa·s HPMC and water system. (**D**–**F**) Separation phase diagrams of SGA and 100, 15,000, and 100,000 mPa·s HPMC and water system.

Cloud point identification involved macroscopic observation of phase-separated systems following centrifugal treatment (4000 rpm, 60 min). Persistent layer differentiation post-centrifugation served as the criterion for phase separation. Figure 2 presents the critical concentration boundaries derived from cloud point data, where the triangular region below the curve represents phase separation conditions, while the rectangular area above corresponds to miscible states. Increased HPMC viscosity resulted in a proportional expansion of phase separation domains for both CGA and SGA, indicating viscosity-dependent enhancement of phase segregation mechanisms.

### 3.3. Viscosity-Driven AGP Enrichment Mechanics

**Figure 3:**
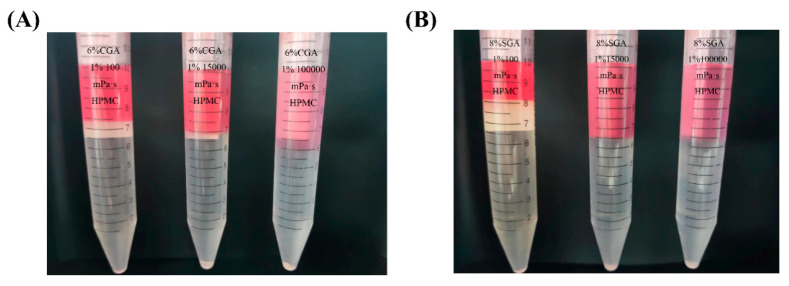
(**A**) Phase separation in the CGA/HPMC mixtures with different viscosities and a fixed concentration ratio of 6% CGA/1% HPMC. (**B**) Phase separation in the SGA/HPMC mixtures with different viscosities and a fixed concentration ratio of 8% SGA/1% HPMC. (CGA, SGA/100 mPa·s, CGA, SGA/15,000 mPa·s, and CGA, SGA/100,000 mPa·s [from left to right]).

The mixtures of CGA (or SGA) and HPMC of various viscosities were prepared as described in experimental Section 2.3. Figure 3 shows the phase separation images of CGA (SGA) and HPMC with different viscosities. The GPC-MALLS analysis determined that in both the CGA/HPMC and SGA/HPMC systems, the upper phase is HPMC rich phase, while the lower phase is the CGA or SGA rich phase. The volume of the upper phase increased with increasing HPMC viscosity, whereas that of the lower phase showed the opposite trend [32].

**Figure 4:**
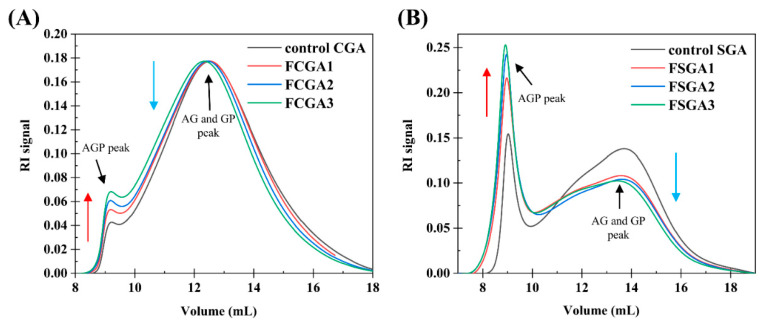
(**A**) RI profiles based on the GPC-MALLS analysis of control CGA and a series of AGP-rich CGA products. (**B**) RI profiles based on the GPC-MALLS analysis of control SGA and a series of AGP-rich SGA products. A series of AGP-rich CGA and SGA products were obtained through varying degrees of phase separation from HPMC. Note: control CGA (or SGA): CGA (or SGA) without phase separation; FCGA1: 6% CGA and 1% 100 mPa·s HPMC blend; FCGA2: 6% CGA and 1% 15,000 mPa·s HPMC blend; FCGA3: 6% CGA and 1% 100,000 mPa·s HPMC blend; FSGA1: 8% SGA and 1% 100 mPa·s HPMC blend; FSGA2: 8% SGA and 1% 15,000 mPa·s HPMC blend; FSGA3: 8% SGA and 1% 100,000 mPa·s HPMC blend.

Figure 4 shows the typical GPC-MALLS curves of the RI signals of the CGA- or SGA-enriched phases in the mixtures containing 1% HPMC of different viscosities and 6% CGA or 8% SGA, respectively. Clearly, the AGP peak was highest at about 9 mL and rose with increasing HPMC viscosity. The AG and GP peaks at approximately 12.5 mL (13.5 mL for the SGA system), on the other hand, decreased with increasing HPMC viscosity. The control CGA and SGA curves in Figure 4A,B revealed that the AGP peak increased and the AG and GP peaks decreased with increasing HPMC viscosity [12,32]. Therefore, the degree of fractionation of CGA and SGA at fixed concentrations rose with the increase in the viscosity of HPMC [33]. In addition to viscosity-driven sieving, the increase in the thermodynamic incompatibility between HPMC and GA at higher viscosities plays a crucial role in enhancing AGP enrichment. This phenomenon results the viscosity of the substances strongly influenced the effect of the phase separation mixture on molecular fractionation at a fixed concentration ratio. Therefore, to concentrate a functional component of one substance, increasing the viscosity of the other can enhance fractionation. Increasing the viscosity of HPMC and mixing it with CGA or SGA led to a higher AGP content. The main reason for this phenomenon is the depletion model effect. When polymers are larger in diameter than the distance between two colloids, polymers in the overlapping region will either be consumed or expelled from this area, imposing surface osmotic pressure on these colloids [1]. In the CGA or SGA enriched phase, the addition of HPMC causes the AG and GP components to be expelled to the HPMC enriched phase. This effect becomes increasingly pronounced with higher HPMC viscosity.

The molecular properties of phase-separated CGA and SGA were analyzed using GPC-MALLS measurements, as described above, to detect the changes in the molecular weights of phase-separated GA as well as the changes in AGP content [34]. Table 2 lists the molecular weights and other characteristics of control and mixed samples of different viscosities.

According to the data, the degree of phase separation (VU/VL, upper and lower phase volume ratio), the Mw of FCGA and FSGA, and the AGP content in FCGA and FSGA increased significantly with the increase in HPMC viscosity. The molecular weight of FCGA was the highest (0.64 × 10^6^ Da) in the 100,000 mPa·s system, which had the highest HPMC viscosity. This value was higher than the molecular weight of control CGA (0.52 × 10^6^ Da). Meanwhile, the molecular weight of FSGA was the highest (5.81 × 10^6^ Da) in the 100,000 mPa·s system, almost twice as high as that of the control SGA (2.82 × 10^6^ Da). At the same time, the contents of AGP in FCGA and FSGA increased from 6.3% and 27.3% in the control to 8.5% and 36.5% in the 100,000 mPa·s HPMC system, respectively. The molecular weight and AGP content of the GA-enriched phase increased after phase separation, in line with the GPC-MALLS findings [12,35]. In addition, the molecular weight of AGP in FCGA and FSGA did not show a significant increase. This indicated that the increase in the molecular weight of FCGA and FSGA was mainly due to the increase in AGP content rather than the aggregation of AGP molecules and a change in chemical composition.

**Figure 5:**
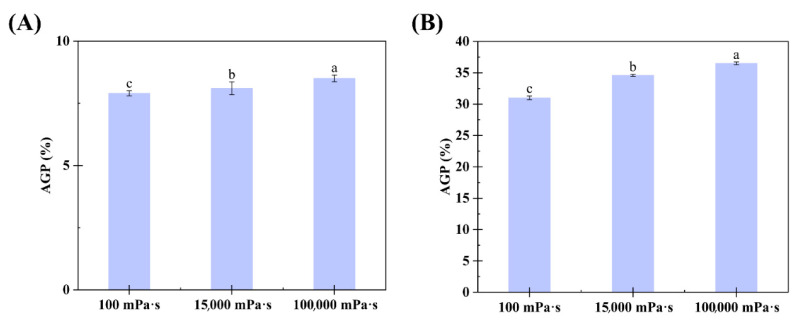
(**A**) Relationship between the AGP content in the lower phase and HPMC viscosity in CGA/HPMC mixtures. (**B**) Relationship between the AGP content in the lower phase and HPMC viscosity in SGA/HPMC mixtures. The letter on each column means the significant difference.

The AGP content in the CGA- and SGA-enriched phases of each formulation was calculated using GPC-MALLS software (version 4.90.08, Wyatt Technology Corporation, USA). The AGP content in the CGA and SGA phases increased significantly with the viscosity of HPMC, from 7.9% and 31% in the 100 mPa·s HPMC system to 8.5% and 36.5% in the 100,000 mPa·s HPMC system, respectively, as shown in Figure 5A,B. This was consistent with the color changes and GPC-MALLS RI curves observed in previous experiments. Thus, the findings showed that viscosity can affect the degree of fractionation in GA, with the tendency of AGP to enter the CGA- or SGA-enriched phase increasing with a rise in HPMC viscosity. When HPMC of the lowest viscosity (100 mPa·s) was used, the AGP level in the CGA-enriched phase (7.9%) was similar to that in the control CGA (6.3%) solution. In contrast, under these conditions, the AGP level in the SGA-enriched phase (31%) was much higher than that in the control SGA solution (27.3%). This could be because the molecular weight of SGA was much higher than that of CGA, and the molecular weight is also a key determinant of the degree of phase separation [36].

### 3.4. Rheology

**Figure 6:**
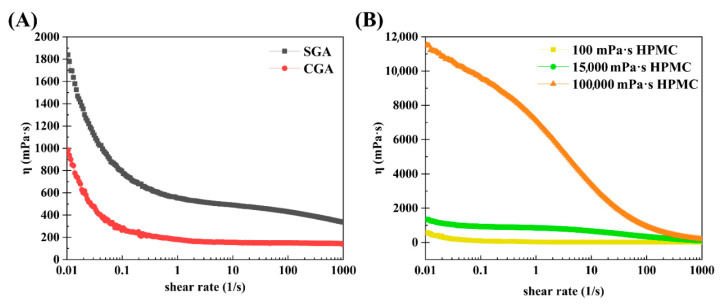
Steady shear viscosity as a function of the shear rate in SGA, CGA, and HPMC. (**A**) Steady-state shear curves of SGA and CGA. (**B**) Steady-state shear curves of HPMC at different viscosities.

The steady-state shear method described in Section 2.7 was employed to determine how viscosity affects the shear rate of CGA, SGA, 100 mPa·s HPMC, 15,000 mPa·s HPMC, and 100,000 mPa·s HPMC. Figure 6A shows the steady-state shear flow curves of CGA and SGA in water at fixed concentrations of 30%. Each of the flow curves demonstrated a shear thinning phenomenon at lower shear rates, and a viscosity plateau was observed at higher shear rates. SGA had a higher viscosity than CGA at the same shear rate due to its higher Mw and Rg [37]. Figure 6B shows the steady-state shear flow curves of the three different HPMC solutions in water, each with a fixed concentration of 1% and different viscosities. Notably, HPMC also exhibited shear thinning at all three viscosities. The flow curves of 100 mPa·s HPMC and 15,000 mPa·s HPMC demonstrated a very low viscosity curve at the same concentration. That was due to the low molecular weight and polymer structure [38,39].

**Figure 7:**
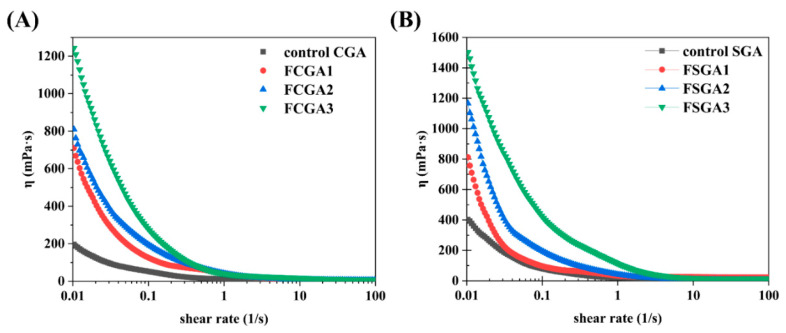
Steady-state shear viscosity as a function of shear rate for GA-enriched phases after the phase separation of CGA (**A**) and SGA (**B**) from HPMC with different viscosities. Note: FCGA1: 6% CGA and 1% 100 mPa·s HPMC blend; FCGA2: 6% CGA and 1% 15,000 mPa·s HPMC blend; FCGA3: 6% CGA and 1% 100,000 mPa·s HPMC blend; FSGA1: 8% SGA and 1% 100 mPa·s HPMC blend; FSGA2: 8% SGA and 1% 15,000 mPa·s HPMC blend; FSGA3: 8% SGA and 1% 100,000 mPa·s HPMC blend.

The shear viscosity values of the control CGA and SGA and the enriched CGA and SGA phases after phase separation were also analyzed (Figure 7). The original CGA and SGA and the phase-separated CGA and SGA samples were consistently adjusted to a final concentration of 6% for testing (as described in Section 2.7). With the change in HPMC viscosity, the rheological behavior was also significantly altered. The shear rate values of the phase-separated CGA and SGA samples were highly correlated with the viscosity of HPMC, and the flow curves were consistent with shear thinning behavior [24]. However, the samples had different viscosity values. FCGA (or FSGA) also tended to show an initial increase in viscosity with increasing HPMC viscosity. The higher viscosity of the GA samples after phase separation could be attributed to the increase in AGP content and the molecular weight of FCGA (or FSGA) following the increase in HPMC viscosity during phase separation. Notably, this molecular weight was an important factor affecting the increase in the viscosity of FCGA (FSGA) during segregative phase separation [40]. Meanwhile, a small amount of HPMC remained in the GA-enriched phase due to the presence of HPMC during phase separation, and this residual HPMC could also contribute to the higher viscosity of FCGA (or FSGA). It has been shown that the increase in viscosity of the HPMC phase correlates directly with the enhanced molecular fractionation observed in the GA/HPMC systems. This effect is largely attributed to the suppression of molecular diffusion, which slows down the redistribution of polymer components and leads to more efficient separation.

## 4. Conclusions

Experimental results demonstrated that both CGA and SGA exhibit distinct phase-separation characteristics when blended with HPMC solutions of varying viscosities (100, 15,000, and 100,000 mPa·s). Modulation of HPMC viscosity effectively influences the dynamics of GA/HPMC systems, resulting in pronounced molecular fractionation effects and elevated AGP concentrations in FCGA and FSGA derivatives. Higher viscosity levels within specified parameters enhance phase segregation efficiency, leading to improved molecular weight differentiation and increased AGP yields. These physicochemical alterations may potentially improve GA’s emulsification properties and expand its functional utility in food systems. The study provides new insights into viscosity-driven fractionation mechanisms in polydisperse GA/HPMC mixtures, which could guide the selection of alternative biopolymers for multiphasic separation studies. Additionally, the findings establish a systematic framework for evaluating multifactorial impacts on molecular partitioning during phase-separation processes.

## Figures and Tables

**Table 1:** Molecular weight characterization of CGA, SGA, and the three types of HPMC solutions.

Sample	Signals	Mw (10^5^ Da)	Polydispersity (Mw/Mn)	Rg (nm)	Mass Recovery (%)
CGA	Peak 1 (AGP)	15.15 ± 0.10 ^c^	1.06 ± 0.05 ^e^	24.20 ± 0.46 ^f^	6.30 ± 0.07
	Peak 2 (AG + GP)	3.76 ± 0.09 ^ef^	1.47 ± 0.04 ^cde^	9.13 ± 0.10 ^i^	93.70 ± 0.23
	Peak 3 (whole gum)	5.23 ± 0.31 ^d^	1.66 ± 0.09 ^bcd^	12.68 ± 0.08 ^h^	100.00
SGA	Peak 1 (AGP)	66.01 ± 0.27 ^a^	1.79 ± 0.10 ^bc^	57.11 ± 0.15 ^c^	27.30 ± 0.17
	Peak 2 (AG + GP)	3.87 ± 0.11 ^e^	2.10 ± 0.60 ^b^	22.40 ± 0.10 ^g^	72.70 ± 0.12
	Peak 3 (whole gum)	28.23 ± 0.47 ^b^	8.41 ± 0.18 ^a^	31.43 ± 0.13 ^e^	100.00
100 mPa·s HPMC	-	0.73 ± 0.06 ^g^	1.46 ± 0.13 ^cde^	34.71 ± 0.17 ^d^	-
15,000 mPa·s HPMC	-	3.33 ± 0.21 ^f^	1.35 ± 0.08 ^cde^	66.61 ± 0.14 ^b^	-
100,000 mPa·s HPMC	-	5.88 ± 0.17 ^d^	1.23 ± 0.06 ^de^	87.65 ± 0.13 ^a^	-

Note: Mw, Weight average molecular weight; Rg, radius of gyration. Values are means of three determinations (n = 3). Different letters indicate significant difference (*p* < 0.05) comparing the mixtures within the same column.

**Table 2:** Phase volume measurements for CGA + HPMC and SGA + HPMC mixtures of different viscosities.

Mixture	V_U_/V_L_	Mw of FCGA/FSGA (×10^6^ Da)	AGP in FCGA/FSGA (%)	Mw of AGP in FCGA/FSGA (×10^6^ Da)
Control CGA	-	0.52 ± 0.03 ^f^	6.30 ± 0.13 ^f^	1.52 ± 0.03 ^f^
6% CGA + 1.0% 100 mPa·s HPMC	0.35 ± 0.02	0.64 ± 0.04 ^e^	7.90 ± 0.05 ^e^	1.64 ± 0.05 ^e^
6% CGA + 1.0% 15,000 mPa·s HPMC	0.42 ± 0.06	0.65 ± 0.03 ^e^	8.10 ± 0.06 ^e^	1.65 ± 0.01 ^e^
6%CGA + 1.0% 100,000 mPa·s HPMC	0.51 ± 0.02	0.64 ± 0.06 ^e^	8.50 ± 0.06 ^e^	1.60 ± 0.03 ^ef^
Control SGA	-	2.82 ± 0.06 ^d^	27.30 ± 0.63 ^d^	6.60 ± 0.09 ^c^
8% SGA + 1.0% 100 mPa·s HPMC	0.21 ± 0.04	4.03 ± 0.09 ^c^	31.00 ± 0.53 ^c^	6.88 ± 0.07 ^b^
8% SGA + 1.0% 15,000 mPa·s HPMC	0.51 ± 0.10	5.19 ± 0.16 ^b^	34.60 ± 0.55 ^b^	7.20 ± 0.12 ^a^
8% SGA + 1.0% 100,000 mPa·s HPMC	0.60 ± 0.03	5.81 ± 0.13 ^a^	36.50 ± 0.31 ^a^	6.32 ± 0.13 ^d^

Note: VU/VL, Upper and lower phase volume ratio; Mw, molecular weight; FCGA/FSGA, CGA/SGA after phase separation. Values are means of three determination (n = 3). Different letters indicate significant difference (*p* < 0.05) comparing the mixtures within the same column.

## Data Availability

The original contributions presented in this study are included in the article. Further inquiries can be directed to the corresponding author.
